# MiR-501 promotes tumor proliferation and metastasis by targeting HOXD10 in endometrial cancer

**DOI:** 10.1186/s11658-021-00268-7

**Published:** 2021-05-22

**Authors:** Xiaomei Sun, Lingtong Hou, Chunping Qiu, Beihua Kong

**Affiliations:** 1 Department of Obstetrics and Gynecology, Obstetrics and Gynecology Hospital of Fudan University, Shanghai, People’s Republic of China; 2 Key Laboratory of Gynecologic Oncology of Shandong Province, Qilu Hospital of Shandong University, Jinan, People’s Republic of China; 3 Department of Radiation Oncology, Shanghai General Hospital, Shanghai Jiao Tong University School of Medicine, Shanghai, People’s Republic of China; 4 Department of Obstetrics and Gynecology, Qilu Hospital of Shandong University, 107 Wenhua Xi Road, Jinan, 250012 Shandong People’s Republic of China

**Keywords:** MiR-501, Endometrial cancer, Proliferation, Metastasis, HOXD10

## Abstract

**Background:**

Several studies have shown the crucial role of miR-501 in regulating cellular pathology in various cancers. However, the function and expression of miR-501 in endometrial cancer (EC) remain obscure.

**Methods:**

The expression of miR-501 was determined using quantitative real-time PCR. MTT assay, colony formation assay and cell cycle analysis were used to evaluate the proliferation ability. Migration and invasion were assessed using transwell assay. Tumor formation in nude mice was used to observe the effects of miR-501 on cell proliferation and migration in vivo. Luciferase assay, quantitative real-time PCR and western blot were applied to determine that HOXD10 was the target gene of miR-501.

**Results:**

In this study, we observed significantly up-regulated expression of miR-501 in endometrial cancer, which correlated with higher pelvic lymph node metastasis and shorter overall survival in high-grade endometrial cancer. High expression of miR-501 was also found in the copy-number-high group than other groups. Moreover, in vitro and in vivo assay showed that overexpression of miR-501 can promote proliferation and metastasis. Mechanistically, we found that miR-501 promotes tumor progression by directly targeting HOXD10. Further study also indicated that miR-501 overexpression can activate the AKT/mTOR pathway.

**Conclusions:**

MiR-501, which functions as an oncomir in endometrial cancer, might be a potential therapeutic target in high grade endometrial cancer.

**Supplementary Information:**

The online version contains supplementary material available at 10.1186/s11658-021-00268-7.

## Background

Endometrial cancer is one of the most common female malignancies. According to new statistics, there will be approximately 65 620 cancer cases diagnosed in the United States in 2020, accounting for 7% of all new diagnosed female cancers; the incidence increased by 1.3% per year from 2007 to 2016 and the death rates also rose over the past decade [1]. Though the 5-year survival of early staged EC can be up to 90%, the clinical outcomes worsen dramatically for patients with recurrent or advanced disease and for patients diagnosed with a clinically aggressive histologic subtype, such as high grade endometrioid, papillary serous and clear cell histologies [2, 3]. Previous studies have found that high grade carcinomas account for fewer new diagnoses but represent more deaths, which means that high grade tumors are more likely to recur and present at an advanced stage [4]. However, some patients with similar ECs might have different outcomes, especially seen among high grade EC patients [5]. All the data reflect the need for a more comprehensive understanding of the molecular genetics of endometrial cancer, especially for high grade ECs.

MiRNAs are small non-coding RNAs that regulate gene expression at the post-transcriptional level, which can act as tumor suppressors or tumor inducers [6]. Studies have shown that miRNAs are involved in a variety of biological processes, such as proliferation, autophagy, metastasis, apoptosis and other processes of cancer [7–11]. Several studies have described the function of miRNAs in endometrial cancer [12, 13]. Our previous study also demonstrated that miR-652 plays an important role in tumor progression of EC [14].

HOXD10, a member of the Abd-B homeobox family, encodes a protein with a homeobox DNA-binding domain, which serves as an important transcription factor targeting downstream proteins [15, 16]. It is abnormally expressed in several cancers, such as prostate cancer, esophageal squamous cell carcinoma, endometrial cancer, colon cancer glioblastoma and ovarian cancer [15, 17–21]. HOXD10 has also been verified as the primary effector that negatively regulates metastasis in cancers, and it was demonstrated to have an inhibitory influence on tumor cell proliferation [22].

In this study, we found a prognosis-associated miRNA, miR-501, the high expression of which predicts poor overall survival of patients with high grade endometrial cancer. Moreover, we established that the tumor suppressor gene HOXD10 was the direct functional target of miR-501 in EC. We believe that these results will advance the progress that is being made in the treatment of patients with high grade EC.

## Methods

### Human tissues and cell lines

Endometrial cancer tissues and the benign endometrium samples were collected from Qilu Hospital, Shandong University. A total of 48 endometrial samples were analyzed, including 15 benign endometrial samples and 33 endometrial carcinoma tissues (EC). All malignant cases were diagnosed according to the criteria of the International Federation of Gynecology and Obstetrics (FIGO). The proportions of tumor cells were evaluated by the frozen section before RNA extraction. The study was conducted in accordance with the Declaration of Helsinki. All participants signed informed consent forms according to the protocols approved by the Ethics Committee of Shandong University.

AN3 CA and Ishikawa cell lines were purchased from the American Type Culture Collection (ATCC). HEK293T was purchased from China Type Culture Collection (Shanghai, China). The AN3 CA cell line was cultured in Eagle’s minimum essential medium. Ishikawa and HEK293T cell lines were maintained in DMEM. All cell culture media were supplemented with 10% fetal bovine serum (FBS) and culture medium for AN3 CA cells was also supplemented with 100 mM sodium pyruvate and non-essential amino acid. All cell lines were cultured and maintained in an incubator with 5% CO_2_ at 37 °C.

### Plasmid construction and transfection

The human pre-miR-501 sequence was amplified from normal human genomic DNA and cloned into the pGIPZ lentiviral vector (Open Biosystems) to generate a miR-501 expression vector. Retrovirus solution expressing miR-501 or negative control (NC) was packaged with pMD2G and psPAX2 in HEK293T cells. The solution was supplement with 8 μg/mL polybrene. EC cells (1 × 105) were plated without antibiotics in six-well plates 1 day before incubation for stable transfection. Then the medium was removed and 1 mL of retrovirus solution was added to each well to incubate for 24 h. Then fresh medium containing 2 μg/ml puromycin (Sigma-Aldrich) was added to each well for screening. Cell colonies transfected with lentiviral vector were obtained after 10–14 days of selection.

### Transient transfection

Mimics, inhibitor and the corresponding control (Gene Pharma) of miR-501 were used to obtain the transient up-regulation or down-regulation of miR-501. HOXD10 cDNA was synthesized by Genechem (Shanghai, China). Lipofectamine 2000 (Invitrogen) was used for cell transfection according to the manufacturer’s protocol.

### RNA extraction and real-time quantitative PCR

Total RNA was isolated with TRIzol Reagent (Ambion) following the manufacturer’s instructions. The reverse transcription of miRNA and mRNA was performed using One Step PrimeScript miRNA cDNA Synthesis Kit (Takara) and PrimeScript RT Reagent Kit (Takara), respectively. Quantitative RT-PCR was performed using an SYBR green PCR mix in an Applied Biosystems 7900HT Real-Time PCR System. Primer information is shown in Additional file 4: Table S1.

### Western blot analysis

Cells were lysed in RIPA Lysis Buffer (Beyotin, China) with PMSF (1%). The concentration of protein was measured with a BCA Assay Kit (Thermo Scientific). Protein samples were separated by SDS-PAGE and transferred onto PVDF membranes (Millipore). The membrane was blocked with 5% non-fat milk for 1 h at room temperature and then incubated overnight at 4 °C in the diluted primary antibody solution. After incubation with horseradish peroxidase-coupled secondary antibody for 1 h (Dako, 1:5000), the bands were washed in TBST solution three times. Then the band signals were detected with enhanced chemiluminescence (ECL) (PerkinElmer) using the ImageQuant LAS 4000 system (GE Healthcare Life Sciences). The following antibodies were used: rabbit anti-HOXD10 (Abcam, Ab138508, 1:5000), rabbit anti-mTOR (Abcam, Ab32028, 1:2500), rabbit anti-AKT (Ab18785), rabbit anti-AKT (phospho T308) (Ab38449) and mouse anti-human β-actin antibody (CST, 3700, 1:1000 dilution).

### Cell viability assay

Both MTT assay and colony formation assay were performed to evaluate cell viability in EC cells. For MTT assay, transfected EC cells were seeded in a 96-well plate in quintuplicate for 0–5 days. At the indicated time points, 20 µL of 5 mg/ml MTT (3-(4,5-dimethylthiazol-2-yl)-2,5-diphenyltetrazolium bromide, Sigma-Aldrich) was added to each well. After incubation at 37 °C for 4 h, the supernatants were carefully removed and 100 µL of DMSO was added to each well. Then the plate was shaken gently in the dark for 10 min and the absorbance value was evaluated by a Varioskan Flash microplate reader (Thermo Scientific) at 490 nm.

For colony formation assay, 500 single transfected EC cells were seeded in a 6-well plate in triplicate and incubated in media containing 10% FBS for about 2 weeks. Next, when the colonies were visible, the supernatant was discarded and the plates were washed 3 times carefully with PBS solution, then the colonies were fixed with methanol and stained with crystal violet for 15 min. The stained colonies were washed with running water and counted under a microscope after drying. Only colonies containing more than 50 cells were counted to determine cell colony formation ability.

### Cell cycle

The cell cycle was analyzed using flow cytometry after propidium iodide (PI) staining. Cells were seeded in 6 cm dishes at a density of 2.0 × 10^5^ cells per well and were transiently transfected with miR-501 mimics and a negative control 24 h later. Cells were harvested until they reached 60–70% confluence then re-suspended in ice-cold 70% ethanol. After being fixed in ice-cold 70% ethanol at 4 °C overnight, cells were washed gently 3 times with cold PBS and then treated with PI and 7-AAD staining solution in the dark for 5 min. The cells were analyzed using the BD FACSCalibur Flow Cytometer (BD Biosciences, Franklin Lakes, NJ, USA) for 1 h and the results were analyzed using ModFit LT software (version 2.0; Verity Software House, Topsham, ME, USA). The experiment was performed in triplicate and repeated three times.

### Migration and invasion assays

The invasion and migration abilities were evaluated using Boyden chamber-style cell culture inserts with and without Matrigel respectively (BD Falcon). EC cells were harvested and suspended, then 200 μl of serum-free media containing 1–2 × 10^5^ cells were added to the upper chambers of a transwell system (24-well, 8 μm pore size, BD falcon). The lower chambers were filled with 500 μl of culture media containing 10% FBS as a chemoattractant. After incubation for 12–48 h, the upper chambers were taken out and washed three times with PBS solution then fixed in methanol for 15 min and stained with 0.1% crystal violet for 20 min. The cells on the upper surface were wiped off and the cells that penetrated to the lower surface of the membrane were counted under a light microscope. The experiment was performed in triplicate and repeated three times.

### Tumor formation and metastasis assay in nude mice

Ishikawa cells transfected with pGIPZ-501 or pGIPZ-NC plasmid vector were used in these assays. For tumor formation assays, 4 × 10^6^ cells in 150 μl of PBS were injected subcutaneously into opposite sides of the axilla of the same 4–5-week-old BALB/c nu/nu female mice (NBRI of Nanjing University, China). The size of tumors was measured weekly, and the weight of tumors was measured after these mice were sacrificed.

For metastasis assay in vivo, 1 × 10^6^ cells in 100 ml of PBS were injected into the peritoneal cavity of 4–5-weeks old BALB/c nu/nu female mice. After 3–4 weeks, the mice were sacrificed and examined for growth and metastasis of the tumors in the peritoneal cavity. All the animal experiments were performed with the approval of Shandong University Animal Care and Use Committee (no. TJLAC-020-033; approval date: February 17, 2020).

### Immunohistochemical staining

The fresh tissues were fixed with formalin for at least 24 h and cut into 4-μm-thick sections to evaluate the expression of HOXD10. Tissue slides were deparaffinized in xylene and rehydrated in ethanol. Antigen retrieval was performed at 98 °C by heat-induced epitope retrieval. Primary antibody was then incubated on the slides in a humid chamber overnight at 4 °C. The primary antibody used was anti-HOXD10 (Abcam, Ab85698, 1:200 dilution). Staining was detected with the I-View 3, 3′-diaminobenzidine (DAB) detection system.

### Luciferase reporter assay

The 3’UTR region of HOXD10 was amplified and inserted into the pmirGLO vector (Promega) at SacI and XhoI sites to generate the wild type constructs. The mutant 3’UTR of HOXD10 was generated by overlap-extension PCR. HEK 293 T cells were cultured in 96-well plates 24 h before, and transfected with 50 ng of WT or MT HOXD10 3’UTR constructs and 0.5 pmol miR-501 mimics or negative controls by Lipofectamine 2000 reagent when it reached 70% confluence. After 24 h of transfection, luciferase activity was measured using the Dual-Glo Luciferase Assay System (Promega).

### Statistical analysis

GraphPad Prism 7 (GraphPad Software, USA) was mainly used in data analysis. The SPSS version 25.0 statistical software was used for statistical analyses. Student’s t-test and one-way ANOVA analysis were applied to analyze the statistical differences among different groups. Moreover, p < 0.05 was considered significant (*p < 0.05; **p < 0.01; ***p < 0.001; ****p < 0.0001). For each of the aforementioned independent experiments, data are presented as the mean ± SEM.

## Results

### MiR-501 expression is increased in human EC patients and its high expression predicts poor survival

**Fig. 1:**
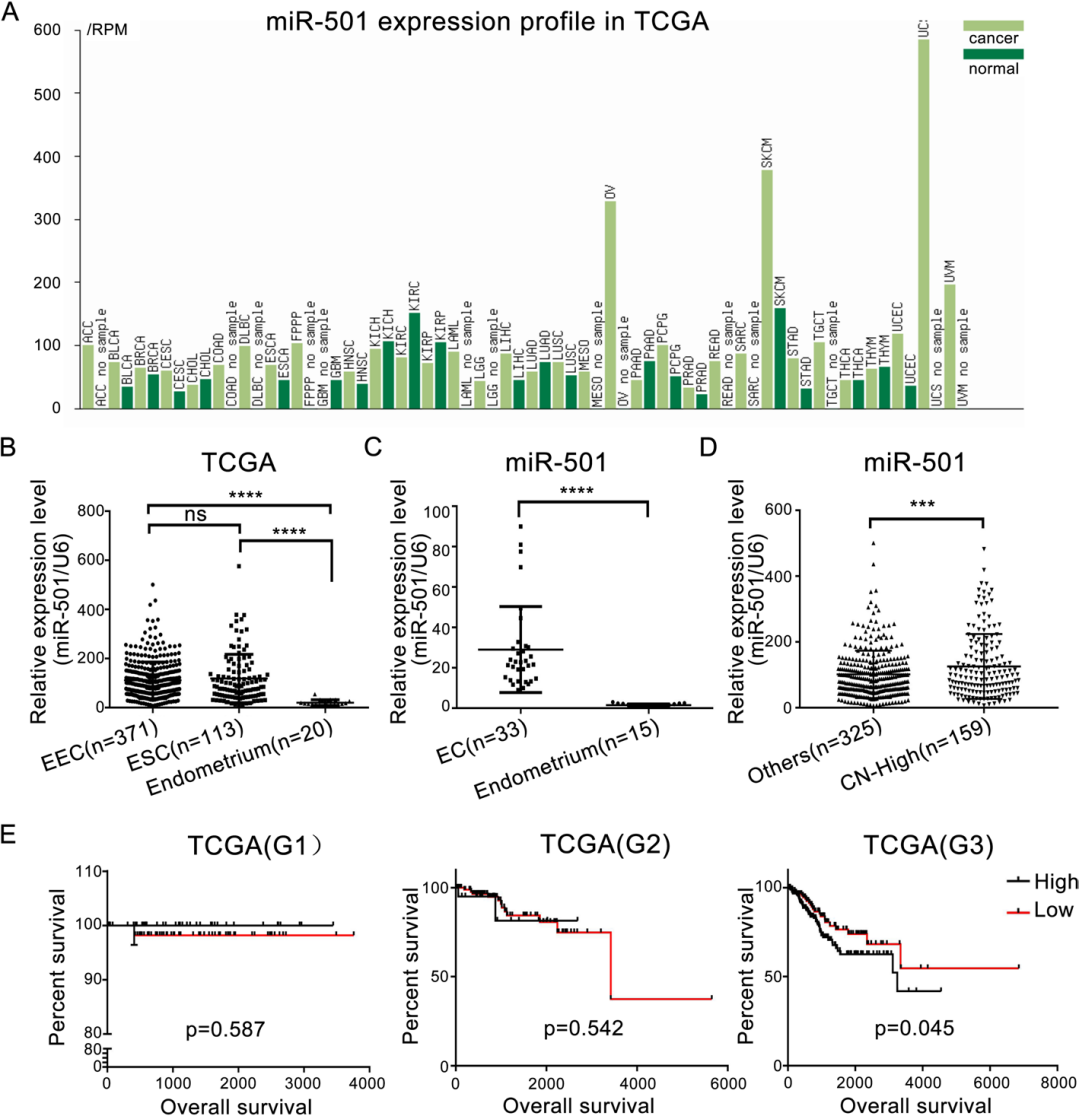
MiR-501 is up-regulated in endometrial cancer and correlates with poor prognosis. **A** Expression profile of miR-501 from TCGA database. MiR-501 showed higher expression in endometrial cancer tissues than normal tissues. **B** Expression of miR-501 was up-regulated in EC tissues compared to the control in the TCGA database. **C** miR-501 was up-regulated in endometrial cancer tissues compared with the control samples (33 EC tissues and 15 benign endometrium tissues). **D** Expression of miR-501 was higher in the CN-high subgroup than other subgroups (data from TCGA). **E** High expression level of miR-501 was associated with shorter overall survival in G3 EC patients (p = 0.045), but no survival difference was found in G1 and G2 EC patients with different miR-501 expression. ***p < 0.001, ****p < 0.0001. G1 low grade; G2 intermediate grade; G3 high grade

In our previous study, we found several miRNAs that showed differential expression between endometrial tissues and benign endometrium, which included miR-501 [14]. To further analyze the expression of miR-501, expression profiles of various types of human cancers and compared tissues were identified from a TCGA data online analysis tool (http://bioinfo.life.hust.edu.cn/miR_path/index.html). The expression of miR-501 was higher in cancer than normal in some tissues, such as bladder urothelial carcinoma, melanoma and endometrial carcinoma. In some other tissues, its expression was higher in normal tissues compared to cancers, such as renal clear cell carcinoma and pancreatic adenocarcinoma (Fig. 1A).

In the TCGA database, miR-501 was up-regulated in EC tissues compared to the control (Fig. 1B). To further determine whether miR-501 was up-regulated in EC, we examined the expression of miR-501 with qRT-PCR (Fig. 1C) and found that miR-501 was significantly up-regulated in EC (n = 33) compared with benign endometrial tissues (n = 15). We also analyzed the expression of miR-501 in the four molecular subtypes in TCGA. The results showed that the miR-501 expression was higher in the copy-number-high (CN-high) group than others (Fig. 1D). We further analyzed the correlation between miR-501 expression and clinicopathological parameters. We found that high miR-501 expression was correlated with pelvic lymph node metastasis (Table 1). TCGA data analysis using the Cox regression test revealed that higher expression level of miR-501 predicted shorter overall survival (OS) in G3 EC patients, but the OS showed no difference in G1 and G2 EC patients (Fig. 1E).

**Table 1:** Correlation between miR-501 expression and EC clinicopathological parameters

Parameters	n	miR-501 level		p value
		Low (n = 332)	High (n = 206)	
Age				
< 55	97	61	36	0.807
≥ 55	437	269	168	
Peritoneal washing				
Negative	344	215	129	0.681
Positive	57	34	23	
Tumor status				
With tumor	77	52	25	0.688
Tumor free	422	275	147	
Lymph nodes (pelvic)				
Negative	339	213	126	0.018*
Positive	73	35	38	
Lymph nodes (aortic)				
Negative	263	160	103	0.119
Positive	36	17	19	
Stage				
I	338	202	136	0.200
II–IV	199	130	69	
Recurrence				
No	415	279	136	0.842
Yes	76	46	30	

The data was from TCGA. The follow-up data of some patients are not complete, so the case number of some groups is smaller than the total, but there is no difference between the expression level of miR-501 in the omitted individuals compared with the listed ones

*p < 0.05

### MiR-501 promotes proliferation of endometrial cancer cells in vivo and in vitro

**Fig. 2:**
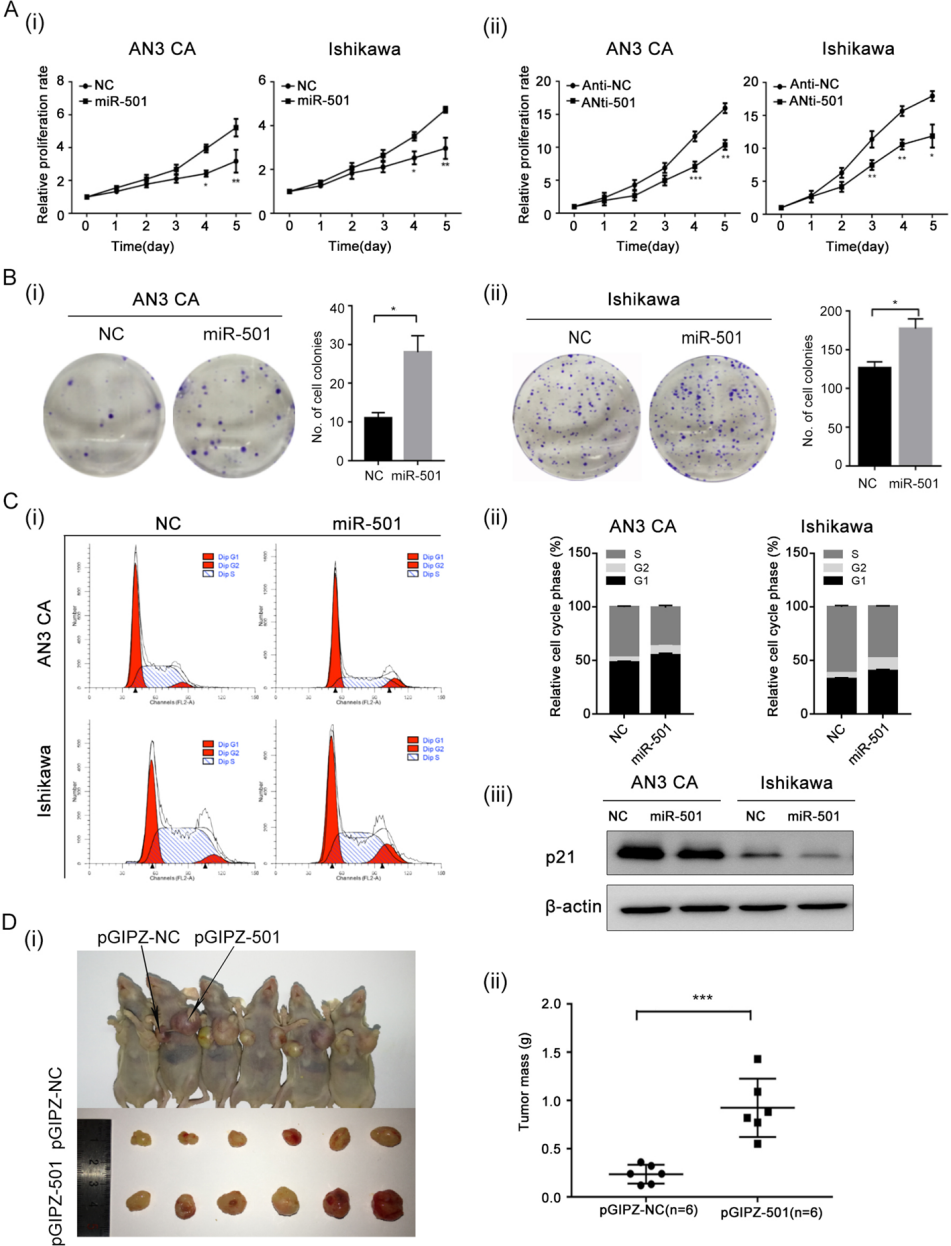
MiR-501 promotes proliferation of endometrial cancer cells. **A** (i) Overexpression of miR-501 promotes cell growth in endometrial cancer cells. (ii) Downregulation of miR-501 inhibited proliferation of endometrial cancer cells. **B** Overexpression of miR-501 could promote colony formation ability in AN3 CA (i) and Ishikawa cell lines (ii). **C** (i, ii) Overexpression of miR-501 can promote the cell cycle into G2 phase. (iii) Upregulated miR-501 can inhibit the expression of p21. D, In nude mice xenograft models, the tumor volume and weight of the miR-501 up-regulated groups were significantly higher than in the control. *p < 0.05, **p < 0.01, ***p < 0.001

Two cell lines with transient miR-501 over-expression and down-regulation were established (Additional file 1: Fig. S1). MTT test and colony formation analysis showed that the up-regulation of miR-501 could promote proliferation and that the cell viability was inhibited when miR-501 was down-regulated (p < 0.05, Fig. 2A, B). The cell cycle assay also showed that the up-regulation of miR-501 can promote the cell cycle into G2 phase and western blot showed that expression of miR-501 was negatively correlated with p21 (Fig. 2C).

To explore the proliferative role of miR-501 in vivo, we also established stably transfected EC cell lines with miR-501 overexpression. The EC cells were transfected with pGIPZ-501 and pGIPZ-NC to establish miR-501 overexpression cell lines and were subcutaneously injected into different sides of the axilla of 4–5-week-old nude mice. Five weeks later, the mice were sacrificed and the tumor was excised to measure the weight and size of the tumor, and it was found that over-expression of miR-501 could obviously promote the growth of EC cells in vivo (Fig. 2D). These assays revealed that miR-501 has the ability to promote proliferation in human EC.

### MiR-501 promotes migration and invasion in endometrial cancer

**Fig. 3:**
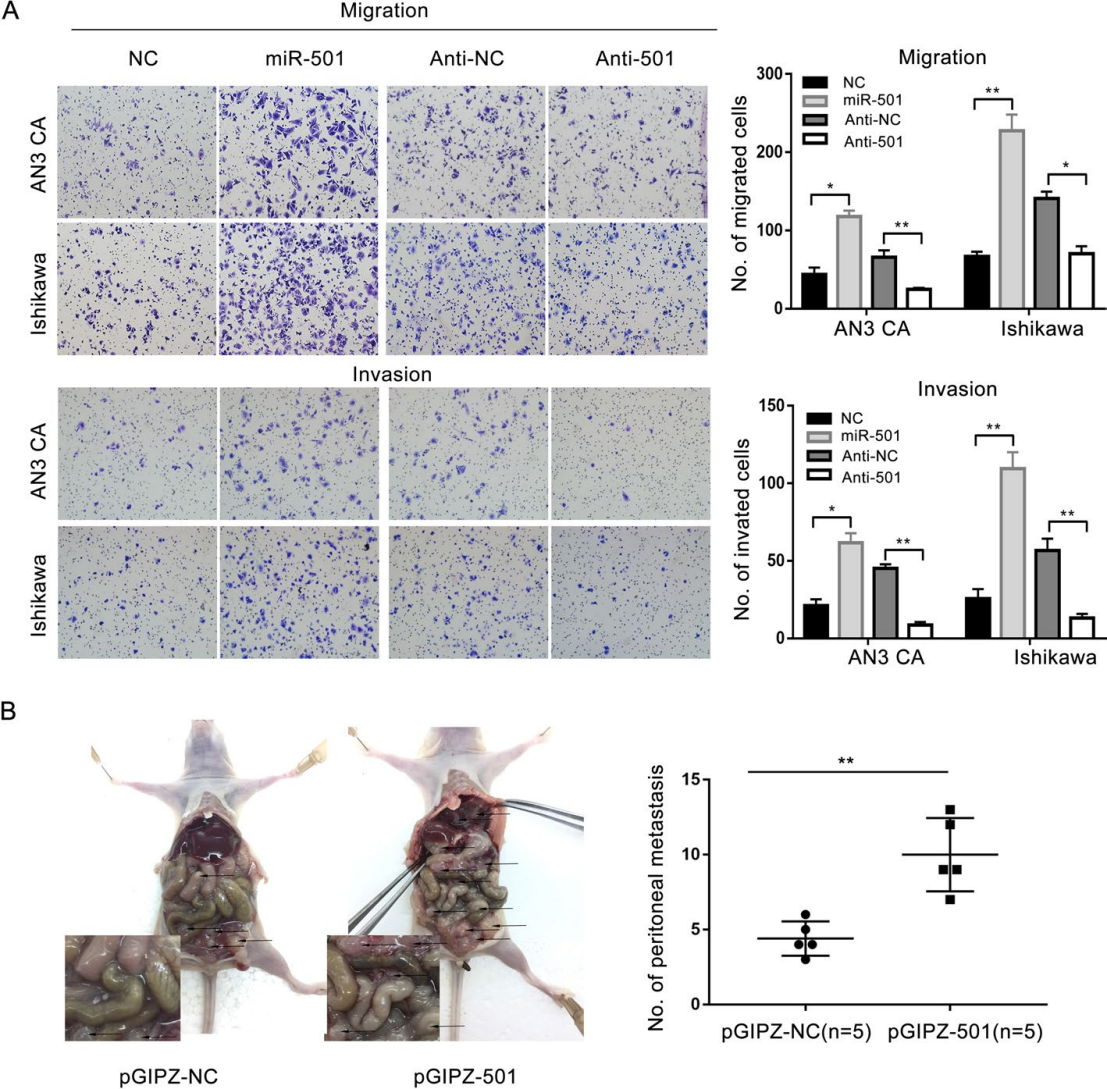
miR-501 promotes migration and invasion of endometrial cancer cells. **A** Upregulation of miR-501 promoted migration and invasion in endometrial cancer cell lines, and downregulation of miR-501 suppressed the migration and invasion ability. **B** The number of peritoneal metastatic nodules in the miR-501 up-regulated group was higher than that in the control group. *p < 0.05, **p < 0.01

In vitro, transwell analysis was conducted to evaluate the metastasis ability of EC cells. The results revealed that overexpression of miR-501 can promote the migrating and invading capacity. The metastatic ability of EC cells was inhibited when miR-501 was downregulated (Fig. 3A). In the in vivo assay, pGIPZ-501 and pGIPZ-NC cell lines were injected into the peritoneal cavities of nude mice. The mice were sacrificed and dissected 4 weeks later, and it was found that the pGIPZ-501 cell group formed more metastatic nodules than the control group (Fig. 3B). All the results showed that miR-501 could markedly promote migration in EC.

### HOXD10 is a direct target of miR-501

**Fig. 4:**
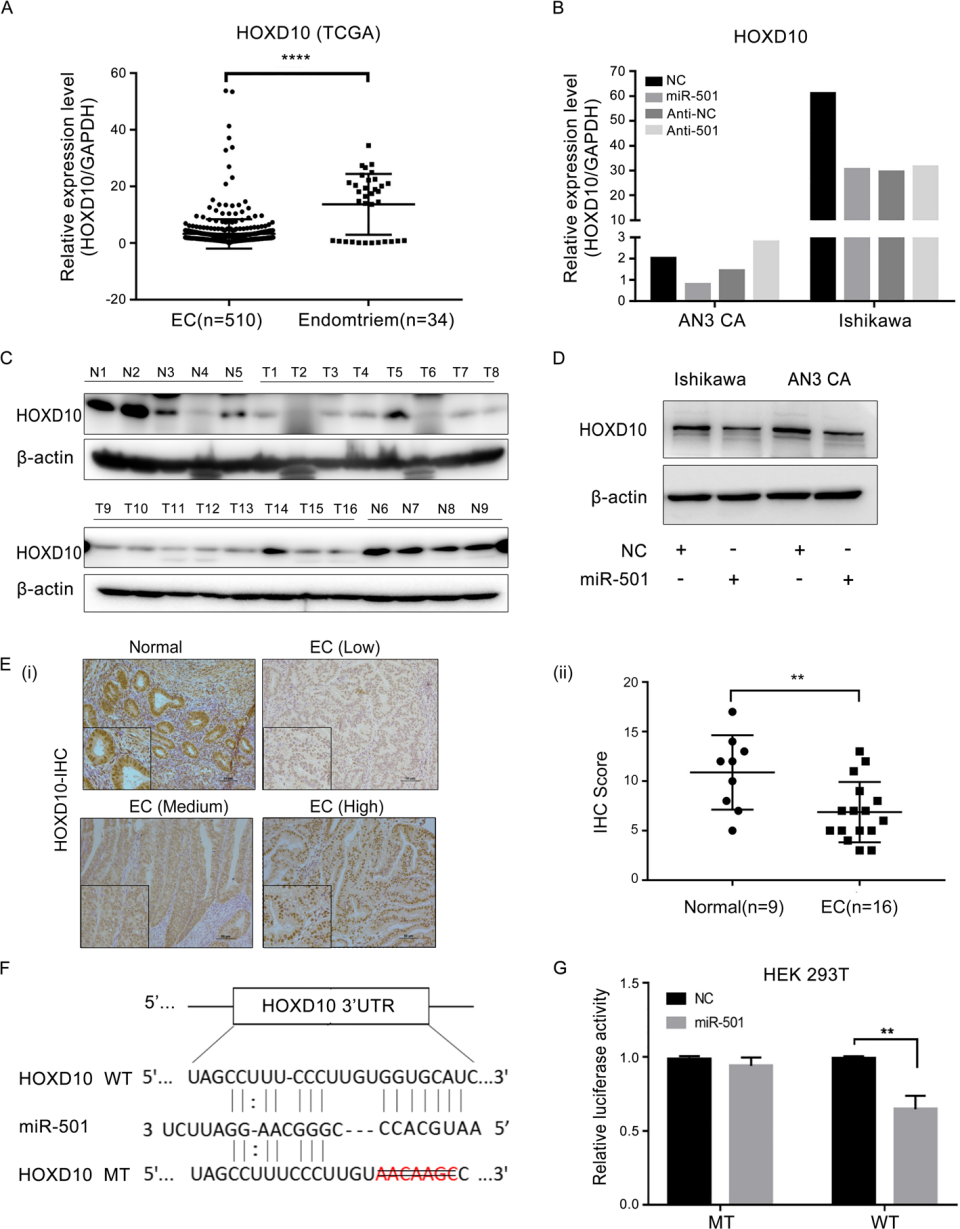
HOXD10 is a direct target of miR-501. **A** At the mRNA level, expression of HOXD10 in endometrial cancer tissues was lower than that in benign endometrial tissue. **B** The mRNA level of HOXD10 was lower in miR-501 up-regulated endometrial cancer cells than in the control. **C** Western blot analysis showed higher protein level of HOXD10 in benign endometrium samples than endometrial cancer tissues (N1–N9 present benign endometrium samples, TI–T16 were collected from endometrial cancer patients). **D** Western blot analysis showed that expression of HOXD10 had an inverse relationship with miR-501 in endometrial cancer cells. **E** Expression of HOXD10 detected by immunohistochemistry. (i) Representative micrograph showing expression of HOXD10 in endometrial cancer tissues and control; (ii) Expression of HOXD10 was higher in benign endometrium than endometrial cancer tissues. **F** Putative binding sequence of miR-501 in the HOXD10 3’UTR, the binding sequence of miR-501 in the HOXD10 3’UTR sequence was deleted to construct the mutant. **G** Overexpression of miR-501 could reduce the luciferase activity when the luciferase reporter plasmid contained WT HOXD10 3’UTR, but no difference was found in the mutant (MT) type. **p < 0.01; ****p < 0.0001

In order to investigate the mechanism by which miR-501 exerts the promotional effect in EC, we need to identify the target gene of miR-501. We analyzed the public databases such as TargetScan, miRNAMap and miRDB and found that HOXD10 was among the predicted target genes. To verify that HOXD10 was the target gene that promotes the development of EC, we first examined the expression of HOXD10 in EC and benign endometrial tissues. In TCGA data, the mRNA level of HOXD10 was higher in benign endometrial tissues compared to that in EC (Fig. 4A). The protein level of HOXD10 was also detected by Western blot (Fig. 4C and Additional file 2: Fig. S2) and immunohistochemistry (IHC) (Fig. 4E). The findings were in accordance with the results obtained from TCGA data.

To detect whether the expression of HOXD10 is regulated by miR-501, HOXD10 expression in miR-501 upregulated and downregulated EC cell lines was tested. The results showed that HOXD10 was negatively correlated with the expression of miR-501 both in mRNA and protein levels (Fig. 4B, D). IHC staining showed that expression of HOXD10 and miR-501 was also negatively correlated in the subcutaneous tumor of nude mice (Additional file 3: Fig. S3). To further confirm that HOXD10 is the direct target of miR-501, we analyzed the sequence of HOXD10, and found the binding site of miR-501 in the 3’UTR of HOXD10 (Fig. 4F). Then the 3’UTR of HOXD10 and the corresponding mutant sequences were introduced into the pmirGLO vector, and the effect of miR-501 on the expression of the target gene was detected using the luciferase analysis. The results showed that the luciferase activity in cells transfected with the wild type 3′UTR of HOXD10 was significantly reduced when miR-501 was over-expressed, but no obvious change was detected in cells with the mutant vector (Fig. 4G). In conclusion, all the results revealed that HOXD10 is directly regulated by miR-501.

### MiR-501 promotes tumor progression via directly targeting HOX10 by activating AKT/mTOR signaling pathway

**Fig. 5:**
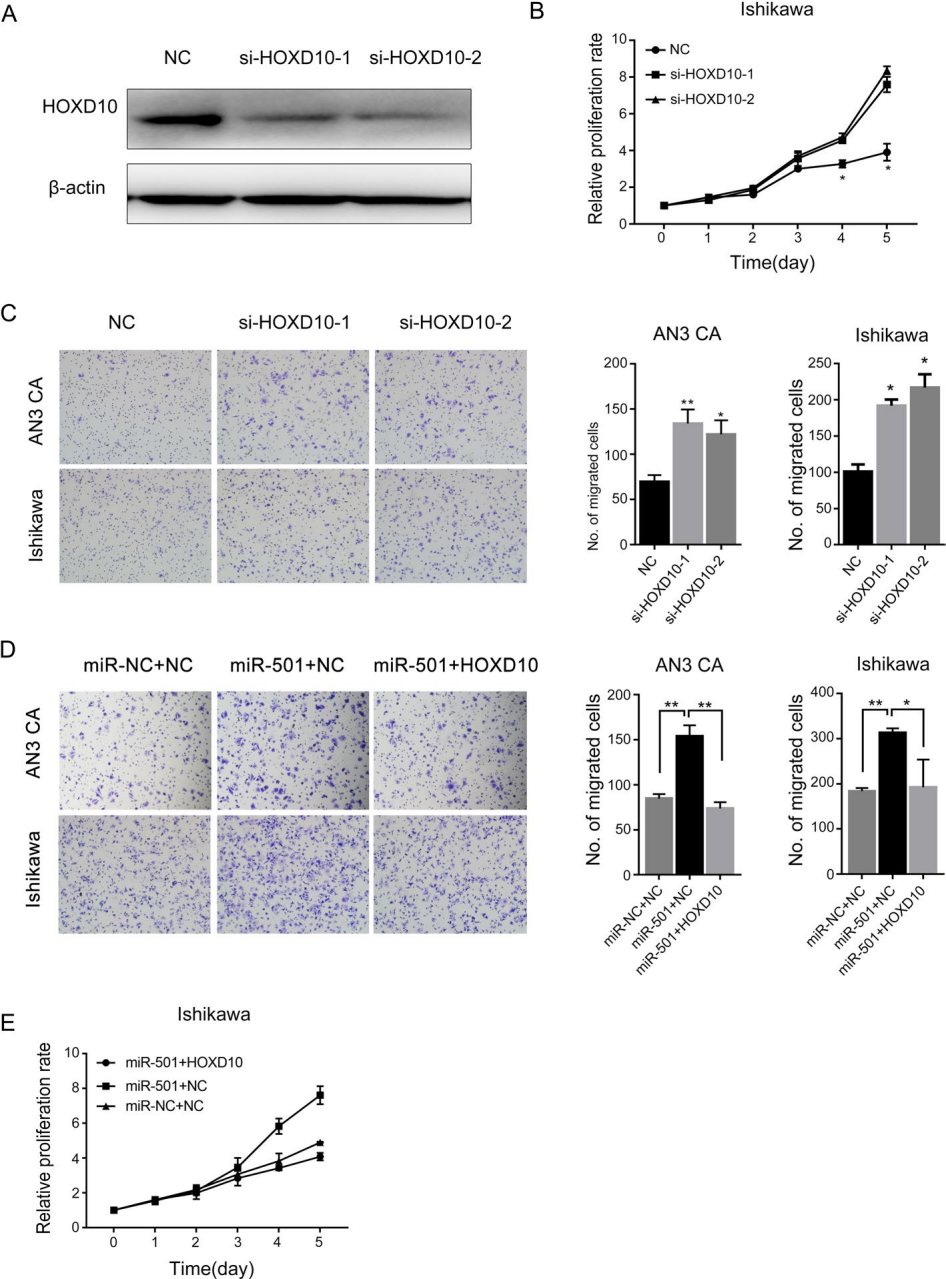
miR-501 promotes endometrial cancer cell growth and metastasis via downregulating HOXD10. **A** Protein levels of HOXD10 after being downregulated using two different siRNAs. **B** MTT assay showed that downregulation of HOXD10 promotes cell growth in Ishikawa cells. **C** Effect of HOXD10 downregulation on migration and invasion in endometrial cancer cell lines. **D** Transwell assay revealed that the migration promoting effect of miR-501 could be rescued by up-regulation of HOXD10. **E** Up-regulation of miR-501 could promote proliferation of Ishikawa cells and over-expression of HOXD10 could abrogate miR-501 induced cell growth. *p < 0.05; **p < 0.01

In order to confirm whether HOXD10 was the direct target gene through which miR-501 promotes EC proliferation and metastasis, we first suppressed the expression of HOXD10 in EC cells (Fig. 5A), and assessed the proliferative and metastatic ability in EC by growth curve and transwell assay. We found that down-regulation of HOXD10 can promote the proliferation and metastasis in EC (Fig. 5B, C). Rescue assay was also conducted to determine whether the promotional effect of miR-501 was achieved through HOXD10. We co-transfected Ishikawa cells with HOXD10 cDNA or negative control and miR-501mimics. The results showed that the promotional effect of miR-501 can be reversed by overexpression of HOXD10 (Fig. 5D, E).

**Fig. 6:**
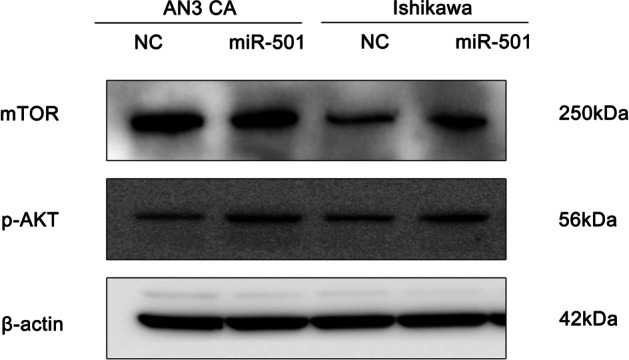
miR-501 promotes tumor progression via miR-501/HOXD10/AKT signaling pathway. Upregulation of miR-501 could increase the expression of p-AKT and mTOR at protein level

In addition, the underlying mechanism of the effect of miR-501 on tumor progression was further explored. We observed increased expression of p-AKT and mTOR following miR-501 overexpression (Fig. 6), which was found to be negatively correlated with HOXD10 expression. Previous studies have confirmed that the AKT/mTOR pathway was associated with the pathogenesis of EC [23]. In conclusion, these results suggest that HOXD10 is one of the main targets through which miR-501 promotes tumor progression in endometrial cancer.

## Discussion

The prognosis of endometrial cancer is influenced by several factors such as stage, histological type, grade, depth of myometrial invasion and nodal status [24]. EC was traditionally classified into two types, type I (estrogen-dependent, with favorable prognosis) and type II (estrogen-independent, with a tendency to recur) [25]. Nonetheless, patients with similar histology may have different outcomes, especially in high-grade EC [26]. In recent studies, molecular typing has also become one of the factors that affect the prognosis of endometrial cancer. The Cancer Genome Atlas (TCGA) project divided EC into four subgroups according to the molecular diversity based on somatic copy number alterations (SCNA) and tumor mutational burden [27]. The four subgroups are POLE-ultramutated, microsatellite instability-hypermutated (MSI-H), copy-number-low (CN-low) and copy-number-high (CN-high). Patients with CN-high EC had a poorer prognosis than others [27]. The National Comprehensive Cancer Network (NCCN) clinical practice guidelines version 2020 in uterine neoplasms recommended molecular analysis in endometrial carcinoma for the first time, which means that the molecular analysis has been implemented in clinical routine.

MiRNA was first described in 1933. Since then, miRNAs have been associated with almost every cell function including proliferation, apoptosis and metastasis [28]. miRNAs are responsible for the regulation of gene expression either by controlling mRNA degradation or by translational repression [29]. Previous studies have demonstrated that miRNAs are associated with disease diagnosis, development and prognosis in several cancers [30]. Several researchers have concentrated on the miRNA profiles using tumor tissues or uterine and blood samples to identify disease-specific biomarkers [31]. MiR-501 was found to promote the progression of several cancers such as colorectal cancer, lung cancer, cervical cancer and gastric cancer [32–35], but the function and mechanism of miR-501 in EC have not been reported.

In this study, for the first time, we found that miR-501 is highly overexpressed in EC tissues compared to the benign endometrial tissues, and the miR-501 expression was higher in CN-high EC tissues than others. High expression of miR-501 is associated with pelvic lymph node metastasis and shorter overall survival. Furthermore, we found miR-501 can promote EC progression by targeting HOXD10. Our mechanistic research also revealed that upregulation of miR-501 can not only inhibit the expression of HOXD10 but also activate the AKT/mTOR pathway. The detection of miR-501 in EC samples is able to distinguish a group of highly aggressive tumors with poor prognosis and to assess the risk of pelvic lymph node involvement.

HOXD10 was found to act as a tumor suppressor, which inhibited the development of esophageal squamous cell carcinoma through the PI3K/AKT/mTOR pathway [21], and in another study, expression of the AKT/mTOR signaling pathway was associated with the progression of EC [36]. In this study, we found that overexpression of miR-501 can inhibit expression of HOXD10 and increase expression of the AKT/mTOR pathway, which was in accordance with the previous studies.

As our understanding about the molecular basis of EC broadens, the identification of novel biomarkers would not only provide more accurate prognostic information but would also aid in improving the clinical management for EC patients. Several clinical trials based on the molecular character have been conducted. A phase II KEYNOTE-158 study demonstrated the clinical benefit of anti-PD-1 therapy among MSI-H/dMMR noncolorectal cancer patients with unresectable or metastatic disease, including EC [37]. In another phase 2 study, lenvatinib and pembrolizumab showed anti-tumor activity in patients with advanced recurrent EC [38]. All the results indicated that precision medicine would be a crucial part of EC therapy.

## Conclusions

In conclusion, we found that miR-501 is overexpressed in CN-high endometrial cancer and proved that miR-501 could promote cell proliferation, invasion and migration in endometrial cancer through directly targeting the downstream target HOXD10. High expression of miR-501 is associated with pelvic lymph node metastasis and poor prognosis of patients with high grade endometrial cancer. These results may provide the basis for further research to determine new diagnoses and treatments for endometrial cancer. Therefore, further studies are required to validate these findings.

## Supplementary Information

**Additional file 1: Figure S1.** Expression of miR-501. A, Expression level of miR-501 in transfected EC cell lines. A, Expression level of miR-501 in two EC cell lines with transient miR-501 upregulation. B, Expression level of miR-501 in two EC cell lines with transient miR-501 downregulation. C, Expression level of miR-501 in two EC cell lines with stable miR-501 upregulation.

**Additional file 2: Figure S2.** Grayscale analysis of the western blot band.

**Additional file 3: Figure S3.** IHC staining showed the expression of HOXD10 in subcutaneous tumor of nude mice.

**Additional file 4: Table S1.** Sequence of primers.

## Data Availability

The datasets used during the current study are available from the corresponding author on reasonable request.

## Abbreviations

EC: Endometrial cancer
HOXD10: Homeobox D10
CN-high: Copy-number high
MSI-H/dMMR: Microsatellite instability-hypermutated/defect mismatch repair
AKT: AKT serine/threonine kinase 1
mTOR: Mechanistic target of rapamycin kinase
NCCN: The National Comprehensive Cancer Network
UTR: Untranslated region
miR-501: MicroRNA-501
