# Supplementary figures and images for: MiR-501 promotes tumor proliferation and metastasis by targeting HOXD10 in endometrial cancer
Source: Cell Mol Biol Lett. 2021 May 22;26:20. doi: 10.1186/s11658-021-00268-7 (PMC8141179; doi:10.1186/s11658-021-00268-7)

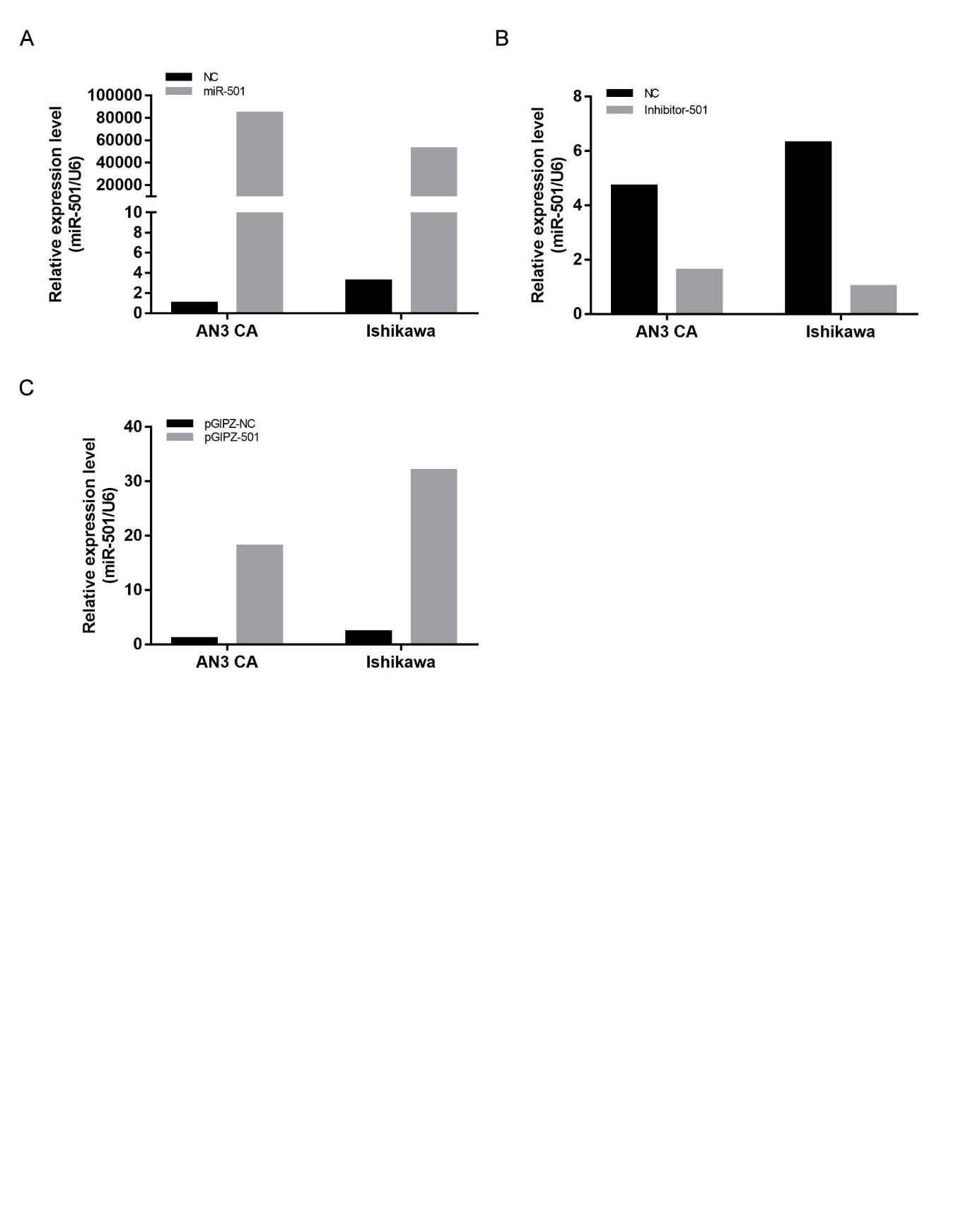

Supplement: Supplementary file 1 — Additional file 1: Figure S1. Expression of miR-501. A, Expression level of miR-501 in transfected EC cell lines. A, Expression level of miR-501 in two EC cell lines with transient miR-501 upregulation. B, Expression level of miR-501 in two EC cell lines with transient miR-501 downregulation. C, Expression level of miR-501 in two EC cell lines with stable miR-501 upregulation. [file 11658_2021_268_MOESM1_ESM.docx]

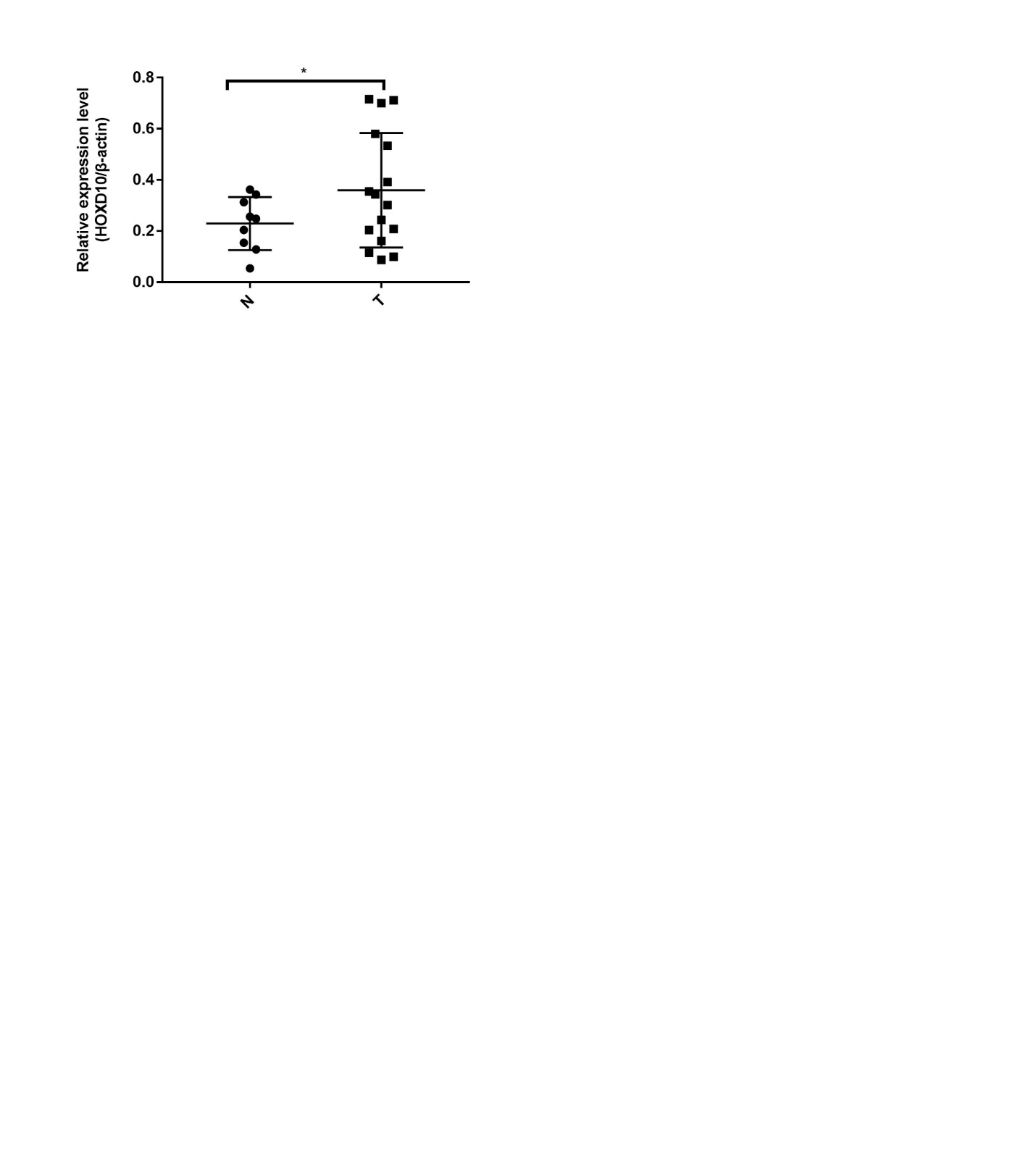

Supplement: Supplementary file 2 — Additional file 2: Figure S2. Grayscale analysis of the western blot band. [file 11658_2021_268_MOESM2_ESM.docx]

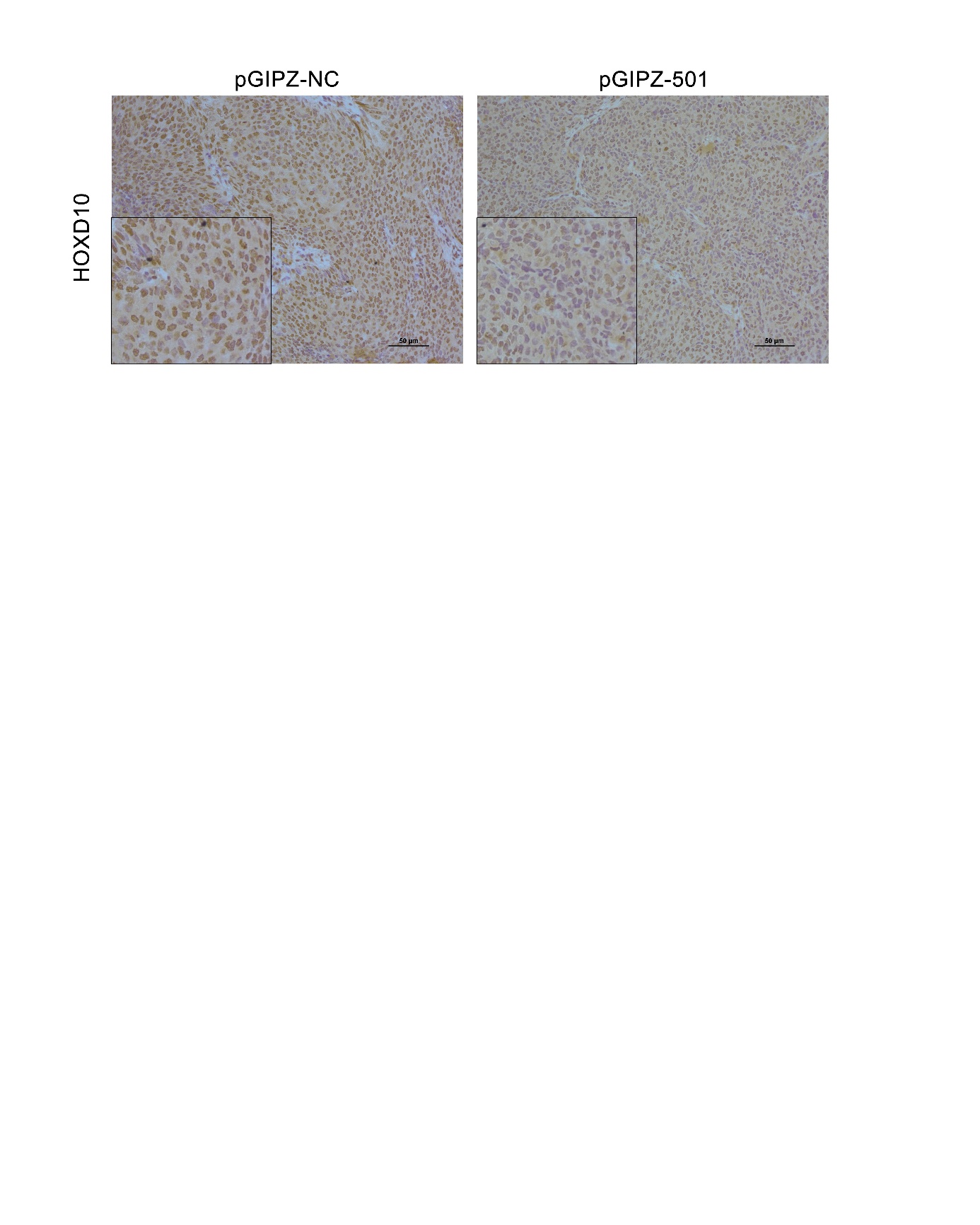

Supplement: Supplementary file 3 — Additional file 3: Figure S3. IHC staining showed the expression of HOXD10 in subcutaneous tumor of nude mice. [file 11658_2021_268_MOESM3_ESM.docx]
