# Supplementary material for: MiR-501 promotes tumor proliferation and metastasis by targeting HOXD10 in endometrial cancer
Source: Cell Mol Biol Lett. 2021 May 22;26:20. doi: 10.1186/s11658-021-00268-7 (PMC8141179; doi:10.1186/s11658-021-00268-7)
Supplement: Supplementary file 4 — Additional file 4: Table S1. Sequence of primers. [file 11658_2021_268_MOESM4_ESM.doc]

Table SI: Sequence of primers

| Method | Primer Name | Primer sequence(5'to3') |
| --- | --- | --- |
| qPCR | U6(F) | TGGAACGCTTCACGAATTTGCG |
| qPCR | U6-R(R) | GGAACGATACAGAGAAGATTAGC |
| qPCR | miR-501(F) | AATGCACCCGGGCAAGGATTCT |
| qPCR | HOXD10(F) | GACATGGGGACCTATGGAATGC |
| qPCR | HOXD10(R) | CGGATCTGTCCAACTGTCTACT |
| qPCR | GAPDH(F) | GGAGCGAGATCCCTCCAAAAT |
| qPCR | GAPDH(R) | GGCTGTTGTCATACTTCTCATGG |
| Construction | GIPZ-puro-miR-501(F) | CCCGTTAACTTGTCTGAGAGGGCATGTCC |
| Construction | GIPZ-puro-miR-501(R) | CCGCTCGAGAGGGGCAATGAGTGGTGAC |
| Construction | HOXD10 3'UTR-WT(F) | CGAGCTCGTCTGAGGCCGGTCTGAGGC |
| Construction | HOXD10 3'UTR-WT(R) | CCGCTCGAGTGAACTCATTTCCAGAGTTTA |
| Construction | HOXD10 3'UTR-MT(F) | CCCTTGTAAGAACGCTGTGGTTT |
| Construction | HOXD10 3'UTR-MT(R) | AAACCACAGCGTTCTTACAAGGG |
